# Higher Household Income and the Availability of Electronic Devices and Transport at Home Are Associated with Higher Waist Circumference in Colombian Children: The ACFIES Study

**DOI:** 10.3390/ijerph110201834

**Published:** 2014-02-07

**Authors:** Diego Gómez-Arbeláez, Paul A. Camacho, Daniel D. Cohen, Katherine Rincón-Romero, Laura Alvarado-Jurado, Sandra Pinzón, John Duperly, Patricio López-Jaramillo

**Affiliations:** 1 Dirección de Investigaciones, Fundación Oftalmológica de Santander—FOSCAL, Calle 155 A # 29–13, Torre Milton Salazar, Primer Piso, Floridablanca, Colombia; 2 Facultad de Salud, Universidad de Santander—UDES, Bucaramanga, Colombia; 3 Escuela de Medicina, Universidad de los Andes, Bogota, Colombia

**Keywords:** childhood obesity, abdominal obesity, waist circumference, electronic devices, household income, socioeconomic status, Colombia

## Abstract

*Background*: The current “epidemic” of childhood obesity is described as being driven by modern lifestyles with associated socioeconomic and environmental changes that modify dietary habits, discourage physical activity and encourage sedentary behaviors. *Objective*: To evaluate the association between household income and the availability of electronic devices and transport at home, and the values of waist circumference (WC), as an indicator of abdominal obesity, in children and adolescents from Bucaramanga, Colombia. *Methods*: Cross-sectional study of public elementary and high school population, of low-middle socioeconomic status. *Results*: A total of 668 schoolchildren were recruited. After adjusting for potential confounders, significant positive associations between waist circumference and higher household income (*p* = 0.011), and waist circumference and the availability of electronic devices and transport at home (*p* = 0.026) were found. *Conclusions*: In low-middle socioeconomic status schoolchildren in a developing country, those from relatively more affluent families had greater waist circumference, an association that is opposite to that observed in developed countries. This finding could be related to higher income family’s ability to purchase electronic devices and motorized transport which discourage physical activity and for their children to buy desirable and more costly western fast food.

## 1. Introduction

The high prevalence of obesity among children is an internationally recognized public health concern [1]. Obesity among children has immediate adverse effects on quality of life [2], self-esteem [3] and experience of discrimination [4]. Moreover, childhood obesity can persist into adulthood [5] and increases the risk of cardiovascular and metabolic diseases [6,7], giving rise to an increased healthcare burden. The causes of obesity are multi-factorial, but ultimately depends on an imbalance between energy intake (food consumed) and energy expenditure (basal metabolic rate, diet induced thermogenesis and physical activity) [8]. This issue is of increasing relevance in developing countries, such as Colombia, where a rapid transition to a westernized lifestyle and environment is thought to underlie steep increases in the prevalence of obesity [9,10,11].

Western or modern lifestyles and environments are thought to discourage regular physical activity and encourage sedentary behaviors in children [12]. In particular, sedentary screen-based behaviors, such as computer use and television (TV) watching, may displace physical activity and are independently associated with obesity [13]. Regular physical activity is essential for maintaining muscular function, metabolic homeostasis and cardiovascular health [14,15]. In contrast, physical inactivity seems to promote the development of obesity and pathophysiological sequelae already at a young age, and this fact is of special importance since physical activity in children is also declining [16]. Physical inactivity is associated with overweight in childhood [17] and tracks from childhood into adulthood when it is an independent predictor of premature mortality [18]. Moreover, the spread of western lifestyles in developing countries is also contributing to a high intake of energy-rich foods, animal and vegetable, and refined sugars [9,10].

Socioeconomic status (SES; *i.e.*, household income) is also thought to be an important determinant of obesity [19]. For instance, it has been described that inequalities in nutrition could be related to socio-economic inequalities and household characteristics [20]. While the relationship between SES and obesity in children is inconsistent in developed countries, in developing countries there appears to be a strong positive association between the two [21]. The obesity-SES association could vary by sex, age, and country. However, in general, SES groups with greater access to energy-dense diets (low-SES in industrialized countries and high-SES in developing countries) are at increased risk of being obese than their counterparts [22]. The analysis reported in this paper, as a subanalysis of the ACFIES study, aims to evaluate the cross-sectional association between household income and the availability of electronic devices and motorized transport at home, and the values of waist circumference (WC), as a sensitive marker of abdominal obesity, in children and adolescents from the city of Bucaramanga in Colombia, a developing country.

## 2. Experimental Section

### 2.1. Study Population

During the 2011–2012 school year, we conducted the cross-sectional component of the Association between Cardiorespiratory Fitness, Muscular Strength and Body Composition with Metabolic Risk Factors in Colombian Children (ACFIES) study to identify the prevalence and associations of cardiovascular risk factors (including glycemic status, lipid profile and inflammatory state, among others), in a sample of schoolchildren from both sexes, enrolled in public elementary and high schools (grades 5 and 6), belonging to a low-middle socioeconomic status (SES 1–3 on a scale of 1–6 defined by the Colombian government). These SES were selected, as these represent the vast majority of the Colombian population. All the recruited participants met the general ACFIES inclusion criteria: age range 8 to 14 years, not having any physical disability and be free of any acute infection lasting less than 2 weeks before the inclusion. A non-probabilistic sampling was used to select the participating schools, and within these schools the entire universe of children who fulfilled the inclusion criteria were invited to participate in the study until the estimated sample size was reached. 

The study protocol was in accordance with the Declaration of Helsinki, and the health research ethics board of the Ophthalmological Foundation of Santander—FOSCAL approved all study procedures. The children expressed their interest in participating in the study, and parents or legal guardians gave written informed consent, before the children were included in the study.

### 2.2. Sample Size

In the ACFIES study, the sample size was based on a calculation of the number of subjects estimated to enable a correlation of 0.40 between handgrip strength and C-reactive protein to be demonstrated [23], with a null hypothesis of 0.27, an alpha value of 0.05 and a power of 0.80. Anticipating potential data loss, an additional 10% of participants were recruited.

### 2.3. Exposures of Interest: Household Income, Electronic Devices and Transport

Data on socioeconomic status and home facilities were assessed by parental questionnaires. Parents were asked to indicate their monthly household income. This information was obtained in Colombian pesos (COP) and for the analysis was converted to U.S. dollars (USD; 1 USD = 1,800 COP). The Colombian minimum monthly wage (MMW) is 328 USD. In this analysis the household incomes were categorized as: low income: <1 MMW; low-middle income: 2 to 3 MMW; and middle income: >3 MMW. 

Parents were also asked to indicate which of the following devices were in their homes: TV, refrigerator, computer and motorized transport (vehicle and/or motorcycle). We analyzed the presence or absence of each of the electronic devices or transport, without consideration for the quantity of each one. These devices were selected on the basis of their likely availability in the population studied and their possible associations with sedentary behaviors and dietary patterns. 

### 2.4. Outcome of Interest: Waist Circumference

All anthropometric measurements were performed by well-trained health workers to minimize coefficients of variation. WC was determined at the middle point between the lower edge of the ribs and the iliac anterior spine. The measurement was made at the end of a normal expiration while the subject stood upright. WC was measured using a measuring tape with spring scale (Ohaus 8004-MA, NJ, USA) and registered in centimeters (cm). 

Other anthropometric measurements were weight and height. Participant’s body weight was measured to the nearest 0.1 kg on an electronic device (Tanita BC544, Tokyo, Japan), in underwear and without shoes, and height was measured to the nearest 0.1 cm using a mechanical stadiometer with platform (Seca 274, Hamburg, Germany). BMI was calculated by dividing body weight by the square of height (BMI = weight (kg)/height^2^ (m)). All measurements were according to the procedures as previously described by Lohman *et al*. [24]. 

### 2.5. Other Covariates

We considered sex, age, pubertal development and cardiorespiratory fitness (CRF) as potential confounders for the hypothesized associations. Pubertal development was assessed by Tanner stage of breast development in girls and testicular volume in boys [25]. CRF was evaluated using the level-1 Yo-Yo intermittent recovery test (YYIRT) [26]. The test consisted of 20-m shuttle runs performed at increasing speeds with 10 seconds of active recovery in a distance of 5-m between runs until exhaustion. The end of the test was considered when the participant twice failed to reach the front line in time (objective evaluation) or he felt unable to complete another shuttle at the dictated speed (subjective evaluation). The total distance covered during the YYIRT level 1 was considered as the test score. CRF is not only a sensitive and reliable measure of habitual physical activity [27], but also a relatively low-cost and useful health indicator [28].

### 2.6. Statistical Analysis

Descriptive statistics were computed for variables of interests, and included mean values and standard deviations of continuous variables and absolute and relative frequencies of categorical factors. Normality of distribution was checked for continuous variables using the Sktest and Shapiro-Wilk test. Wilconcox Rank Sum test was used to investigate the differences in continuous variables. Testing for differences in categorical variables was accomplished using the Pearson’s chi-squared test (Chi2). Moreover, we used robust linear regression models to assess the hypothesized associations. These analyses were adjusted for the potential confounders described above. All statistical analysis was carried out using Stata statistical software, release 11.0 (Stata Corporation, College Station, TX, USA). A *p* < 0.05 was considered statistically significant. 

## 3. Results and Discussion

### 3.1. Results

The results presented in this manuscript are a subanalysis of the main ACFIES study. A total of 668 children and adolescents were recruited, of which 351 (52.5%) were boys. The overall mean age was 11.5 (±1.1) years. Significant differences in height (1.45 ± 0.08 *vs.* 1.44 ± 0.09) (*p* = 0.01) and WC (64.86 ± 9.02 *vs.* 66.92 ± 10.24) (*p* = 0.01) were found between girls and boys, respectively. Demographic and anthropometric data are presented in Table 1.

**Table 1:** Demographic and anthropometric data.

	Total (n = 668)	Girls (n = 317)	Boys (n = 351)
Age (years) ^a^	11.52 ± 1.13	11.52 ± 1.10	11.51 ± 1.16
Anthropometric measures ^a^			
Weight (kg)	40.08 ± 10.07	40.33 ± 9.77	39.86 ± 10.35
Height (m)	1.45 ± 0.09	1.45 ± 0.08	1.44 ± 0.09 ^b^
BMI (kg/m^2^)	18.87 ± 3.61	18.81 ± 3.52	18.93 ± 3.68
Waist circumference (cm)	65.95 ± 9.73	64.86 ± 9.02	66.92 ± 10.24 ^b^
Tanner stage (n-%)			
1	368 (55.1)	149 (47.0)	219 (62.4) ^c^
2	207 (30.9)	109 (34.4)	98 (27.9)
3	78 (11.7)	53 (16.7)	25 (7.1)
Household Income ^a^	559.6 (444.02)	521.8 (363.01)	593.2 (387.3)^ b^
Devices (n-%)			
Refrigerator at home	602 (93.2)	281 (93.1)	322 (93.3)
TV at home	639 (98.5)	295 (97.4)	344 (99.4)
Computer at home	360 (55.8)	159 (52.8)	201 (58.4)
Transport at home	305 (48.8)	131 (44.9)	174 (52.4)

Notes: ^a^ Data are presented as mean ± standard deviation for continuous variables. ^b^ Wilconcox Rank Sum test *p* < 0.01. ^c^ *p* < 0.001 Pearson’s chi-squared test (Chi2). BMI: Body mass index. TV: Television. Transport: Motorized transport (vehicle and/or motorcycle).

The most commonly available device at home was TV (98.5%), while refrigerator, computer and motorized transport were only present in 93.0%, 55.8% and 48.8% of the overall participant’s homes, respectively. A significant positive association between income level and availability of the evaluated electronic devices and transport at homes was observed (Table 2). As shown in Table 2, TV was available in most of the participant’s homes, independently of their income level; therefore it was excluded for convenience from further association analysis. 

After adjusting for potential confounders (age, sex, pubertal development and CRF), using a robust linear regression, significant positive and independent relations between WC and both household income (*p* = 0.011) and availability of electronic devices and transport at home (*p* = 0.026) were found (Table 3). 

**Table 2:** Association between socioeconomic status and the availability of the evaluated electronic devices and transport (vehicle and/or motorcycle) at home.

	Total (n = 626)	Middle Income (n = 194)	Low-Middle Income (n = 297)	Low Income (n = 135)
Refrigerator at home ^a^	573 (93.0%)	188 (97.9%)	271 (93.1%)	114 (85.7%)
TV at home ^b^	608 (98.5%)	192 (100%)	286 (98.3%)	130 (97.0%)
Computer at home ^a^	343 (55.8%)	139 (72.4%)	161 (55.5%)	43 (32.3%)
Transport at home ^a^	291 (48.8%)	117 (62.2%)	132 (47.5%)	42 (32.3%)

Notes: ^a^ *p* < 0.001 Pearson’s chi-squared test (Chi2). ^b^ *p* < 0.05 Pearson’s chi-squared test (Chi2). TV: Television. Transport: Motorized transport (vehicle and/or motorcycle).

**Table 3:** Relationship between waist circumference and both household income and the availability of the evaluated electronic devices and transport (vehicle and/or motorcycle) at home.

Variable	Coef.	Std. Err.	95% CI	*p* Value
Household Income	0.002	0.001	0.0005–0.004	0.011
Devices	1.726	0.773	0.206–3.246	0.026

Notes: Adjusted by sex, age, pubertal development, and cardiorespiratory fitness. Devices: the availability of the evaluated electronic devices and transport at home.

### 3.2. Discussion

Although the present study did not include households in the highest socioeconomic strata (SES 4 to 6), children with greater waist circumference, and consequently a higher susceptibility to present with abdominal obesity, were from relatively more affluent families. There are a number of possible explanations for this finding. The child’s food environment is an important determinant of childhood obesity [29] and food consumption may be limited by its lower availability in the home in families with lower income. 

However, there is an interesting discrepancy between developed and developing countries, and the causal direction of the relationship between SES and obesity is complex [22]. In developed countries, obesity tends to be inversely related to household income. The highest energy-density foods are often the cheapest and diets which include foods with a high added sugar or fat content are more accessible to lower income groups than lower energy density, higher quality diets with a higher proportion of lean meats, fish, fruit and vegetables [30,31]. Meanwhile, high-SES individuals in developing countries seem to be at increased risk of being obese than their counterparts [22], probably due to an increased affinity toward a western type of lifestyle [32]. Children from high SES receive daily allowance (pocket money) to buy lunch and snacks. Because of the brand building efforts by transnational companies that heavily target this age group, fast foods, easily available in the school cafeteria, become their preferred choice [33]. 

SES may influence energy balance not only by determining access to food, but also by affecting patterns of physical activity. In developed countries it is reported that children of high SES families have greater access to alternative activities [34,35] including outdoor entertainment an appealing alternative to increase physical activity levels. On the other hand, due to easy availability of domestic help in developing countries, affluent children may resort to a relatively inactive lifestyle [33]. Moreover, the availability of transport at home (vehicle and/or motorcycle) could discourage active commuting either by walking or bicycling. 

The use of electronic devices has been increasing rapidly worldwide, even in populations of developing countries such as Colombia, part of lifestyle changes which increase participation in non-active leisure time activities [12,13,34,35]. Greater access to electronic media in the home might increase screen time and consequently decrease the physical activity levels [12,13,35], while children in homes without these devices are forced to find other forms of leisure time pursuits. Physical inactivity seems to track from childhood into adulthood, and up to 80% of overweight or obese children remain obese in adulthood with significant impact on adult health [16,36].

The westernized food and physical activity patterns described may promote positive energy balance and result in weight gain. Hence, novel strategies to modify unhealthy nutrition patterns [29,30,31] and to provide popular active alternatives to displace sedentary activities should be adopted [37,38]. 

The results of this study highlight that income and consumer good ownership, factors which influence the family environment, are multi-dimensional contributors to childhood adiposity. In Colombia, a developing country, waist circumference is positively associated with higher household income and greater availability of electronic devices and transport at home. Thus, nutritional education programs, and strategies to increase physical activity and reduce sedentary behavior may be fruitful in preventing overweight and obesity in schoolchildren. Early intervention is required to tackle the problem of childhood obesity. Additionally, the findings highlight the need for broadly based family and community level interventions targeting the social, economic and cultural dimensions of overweight and obesity.

### 3.3. Study Limitations

There are some study limitations that warrant consideration. First, the data analyzed in this present research is cross-sectional; therefore, a causal relationship cannot be inferred. Second, while we asked about the availability of electronic devices and transport at home, we did not ask how much time was spent using them. Further studies including this variable are required. Third, our analysis included low and mid SES schoolchildren and households, but not the highest socioeconomic strata of Colombian society, therefore our results may not be generalisable to this subpopulation. However, these social strata do account for the higher proportion of Colombian society and are representative of the social conditions under which the majority of Latin American youth live.

## 4. Conclusions

The present study, to our knowledge is the first to examine the association between waist circumference and both household income and availability of electronic devices and transport at home in Colombian children. The results from this study demonstrate that both higher household income and the availability of electronic devices and transport at home are positively and independently associated with greater waist circumference among children of low-middle SES.
